# Establishment of a Robust and Simple Corneal Organ Culture Model to Monitor Wound Healing

**DOI:** 10.3390/jcm10163486

**Published:** 2021-08-06

**Authors:** Sandra Schumann, Eva Dietrich, Charli Kruse, Salvatore Grisanti, Mahdy Ranjbar

**Affiliations:** 1 Institute for Medical and Marine Biotechnology, University of Luebeck, Moenkhofer Weg 239a, 23562 Luebeck, Germany; charli.kruse@uni-luebeck.de or; 2 Fraunhofer Research Institution for Marine Biotechnology and Cell Technology, Moenkhofer Weg 239a, 23562 Luebeck, Germany; eva-dietrich@mail.de; 3 Department of Ophthalmology, University of Luebeck, Ratzeburger Allee 160, 23538 Luebeck, Germany; Salvatore.Grisanti@uksh.de (S.G.); eye.research101@gmail.com (M.R.); 4 Laboratory for Angiogenesis & Ocular Cell Transplantation, University of Luebeck, Ratzeburger Allee 160, 23538 Luebeck, Germany

**Keywords:** cornea, wound healing, epithelium, disease model, cell migration

## Abstract

The use of in vitro systems to investigate the process of corneal wound healing offers the opportunity to reduce animal pain inflicted during in vivo experimentation. This study aimed to establish an easy-to-handle ex vivo organ culture model with porcine corneas for the evaluation and modulation of epithelial wound healing. Cultured free-floating cornea disks with a punch defect were observed by stereomicroscopic photo documentation. We analysed the effects of different cell culture media and investigated the impact of different wound sizes as well as the role of the limbus. Modulation of the wound healing process was carried out with the cytostatic agent Mitomycin C. The wound area calculation revealed that after three days over 90% of the lesion was healed. As analysed with TUNEL and lactate dehydrogenase assay, the culture conditions were cell protecting and preserved the viability of the corneal tissue. Wound healing rates differ dependent on the culture medium used. Mitomycin C hampered wound healing in a concentration-dependent manner. The porcine cornea ex vivo culture ideally mimics the in vivo situation and allows investigations of cellular behaviour in the course of wound healing. The effect of substances can be studied, as we have documented for a mitosis inhibitor. This model might aid in toxicological studies as well as in the evaluation of drug efficacy and could offer a platform for therapeutic approaches based on regenerative medicine.

## 1. Introduction

The cornea is a transparent, avascular tissue which forms an important barrier through tight junctions present at its superficial layer [1]. This highly specialized tissue has been used extensively as a model system to study wound healing in the human body as its anatomic location causes the cornea to be subject to abrasive forces and physical damage [2]. Corneal epithelial erosion and ulceration are common ocular injuries resulting from multiple causes such as trauma, chemical burns, infections and refractive surgery [3].

Wound healing of the cornea involves the removal of necrotic cells, infiltration of neutrophils, migration of cells from the wound edge covering the wound, mitosis as well as migration of limbal epithelial stem cells, and differentiation of newly formed epithelial cells to more mature, anterior, stratified epithelial cells to restore a smooth multilayered formation [4]. Epithelial lesions normally resolve within several days, without any fibrotic response. However, wound healing speed might be impacted by various diseases such as diabetes, autoimmune disorders or infection of the tissue. During this period, various eye drops are used to support healing and prevent infection. These agents should not adversely affect re-epithelization and reduce scar tissue formation [3].

Several animal models of corneal wound healing have been developed, which played a major role in understanding the regeneration process and helped in the assessment of potential therapeutic medications. Use of in vitro systems offer a great relief from in vivo experiments on animals, but are not able to mimic the complex interaction of different cell types in a vivid surrounding. Alternatives, such as ex vivo organ culture models, are in demand as they combine the advantages of both. However, most of these models are quite difficult to set up from a technical point of view and reproducibility is therefore also often not given, especially for less experienced experimenters [5].

Here, we report an easy-to-establish ex vivo organ culture model for the evaluation and modulation of corneal wound healing. This model has improved on the development of corneal edema with several modifications and as this model uses globes from porcine cadavers, it is closer to the in vivo condition than in vitro cell cultures. In addition, the tissue is regularly available and easy to obtain. As such, this model might aid in toxicological studies as well as in the evaluation of drug efficacy and could offer a platform for therapeutic approaches based on regenerative medicine.

## 2. Materials and Methods

### 2.1. Preparation and Cultivation of Porcine Corneas

Specimens were prepared as previously described [6]. In brief, the pig eyes used for tissue culture were obtained from a local abattoir. Shortly post mortem the eyes were removed and kept cool (4 °C) in phosphate buffered saline (PBS; Thermo Fisher Scientific GmbH, Waltham, MA, USA). The overall preparation of corneal tissue was performed within 6 h. The eyes were freed of excess muscle and connective tissue and placed in a disinfectant solution (penicillin/streptomycin with amphotericin B (Merck KGaA, Darmstadt, Germany) in PBS) for 10 min. The corneal wound was made by carefully pressing a biopsy punch (4 mm or 8 mm diameter; Kai Europe GmbH, Solingen, Germany) onto the middle of the eye while avoiding too much pressure. The epithelium within the resulting circle was then abraded using a hockey knife, so that a defined defect area (corneal erosion) was created. The corneal tissue with the wound was then cut out completely through a 10 mm biopsy punch (Acuderm, Lauderdale, FL, USA) and with the help of scissors. The specimens were then transferred to a 12-well plate with 1 mL of organ culture medium per well and cultivated in the incubator at 37 °C and 5% CO_2_ for 72 h. The medium was changed daily.

Wound healing of the corneal lesions was observed with a Stemi 2000-C stereomicroscope (Carl Zeiss Microscopy GmbH, Oberkochen, Germany). Various culture media (Table 1) and conditions (Table 2) were evaluated.

### 2.2. Determination of Corneal Wound Area

To observe the epithelial wound area, images of the porcine corneas were taken at regular intervals over 72 h using the Stemi-2000C stereo microscope using the dark-field filter. The wound area was quantified using ImageJ (NIH, Version 1.48b, Bethesda, Rockville, MD, USA). For this purpose, the wound margin was outlined with the mouse (polygon tool) and the size of this area was determined in pixels (measure). The number of pixels was converted to mm^2^ using an internal standard.

### 2.3. Lactate Dehydrogenase Assay for Cytotoxicity Estimation

In order to estimate the tissue integrity and viability of the corneal disks during ex vivo culture the Cytotoxicity Detection Kit PLUS LDH (Roche, Basel, Switzerland) was used. This colorimetric assay quantified cell death and cell lysis, based on the measurement of lactate dehydrogenase (LDH) activity released from the cytosol of damaged cells into the medium. The cell culture supernatants from the organ culture were collected and stored at −20 °C until further use. After thawing the supernatants were centrifuged by 250× *g* and 4 °C for 5 min followed by the transfer of a 100 µL volume per sample in a well of a 96 well plate. The test was subsequently carried out according to the manufacturer’s instructions. The formed red formazan was detected spectroscopically. The absorption of the samples was measured at 492 nm and 690 nm in a microplate reader (CLARIOstar, BMG Labtech GmbH, Ortenberg, Germany). Media samples cultivated without cornea tissues served as controls. The values measured at an absorption of 690 nm represented the background and were subtracted from the measured values at 490 nm. Finally, the mean values of the media controls were subtracted from the difference values of [A_492_–A_690_]. The amount of formazan is proportional to the amount of LDH and thus a measure of cytotoxicity.

### 2.4. Tissue Processing for Paraffin Sections

The porcine corneas were initially placed in embedding cassettes (VWR International GmbH, Darmstadt, Germany). They were fixed in formalin, dehydrated through a series of graded ethanol baths and finally embedded in paraffin (Sigma-Aldrich, Taufkirchen, Germany). Paraffin wax blocks with embedded tissue were created and stored at 4 °C until further processing. The paraffin sections were cut with a thickness of 4 μm using a microtome. The sections were initially stored overnight at 37 °C and until further use at room temperature. Before usage for further analysis (e.g., HE staining) the tissue sections had to be dewaxed. Therefore, the sections were dipped three times for 7 min each in xylene, twice for 3 min each in 100% ethanol and then they were hydrated for 2 min each in a descending alcohol series. Until further use, the sections were placed in distilled water (dH_2_O) for at least 5 min.

### 2.5. Haematoxylin and Eosin Staining of Corneal Sections

After an initial brief rinse in dH_2_O, the corneal paraffin sections were stained for 3 min with Mayer’s hemalum solution (Carl Roth, GmbH + Co. KG, Karlsruhe, Germany). The sections were then briefly washed again in dH_2_O and then blued for 20 min under running tap water. After another washing step in dH_2_O, the samples were stained with eosin solution (Carl Roth, GmbH + Co. KG, Karlsruhe, Germany) for 30 s, washed in dH_2_O and immersed for a short time in solutions of ascending alcohol series (70, 80, 90, 96%). Finally, the sections were covered with Euparal (Carl Roth, GmbH + Co. KG, Karlsruhe, Germany) and left to dry for at least 1 h. Tissue sections were analysed with the Discovery.V8 stereomicroscope (Carl Zeiss Microscopy GmbH, Oberkochen, Germany) and histological overviews were created using the mosaic tool.

### 2.6. Evaluation of Apoptosis with TUNEL Assay

To detect apoptosis in the corneal tissue sections the “In situ Cell Death Detection Kit, TMR red” (Roche, Basel, Switzerland) was used according to the manufacturer’s instructions. This TUNEL (Terminal deoxynucleotidyl transferase dUTP nick end labeling)-Assay is based on the detection of single and double strand breaks in the DNA that occur during early apoptosis. For this method, the paraffin tissue sections were first dewaxed for 30 min in xylene, 5 min in isopropanol and were then rehydrated for 5 min in 70% ethanol and for a short time in PBS. The sections were permeabilized for 8 min with the TUNEL permeabilization solution and then washed twice with PBS. The samples were then incubated for 1 h at 37 °C using the reaction mix containing terminal deoxynucleotidyl transferase (TdT) and tetramethylrhodamine (TMR)-dUTP. The negative control was incubated without a reaction mix, whereas the positive control was incubated with deoxyribonuclease (DNase) I beforehand for 30 min. The samples were then washed three times with PBS following a nuclear staining using 4′,6-diamidino-2-phenylindole (DAPI; Roche, Basel, Switzerland) for 10 min. Finally, sections were covered with Vectashield (Vector Laboratories, Burlingame, CA, USA) and were analysed using the Axio Observer Z.1 fluorescence microscope (Carl Zeiss Microscopy GmbH, Oberkochen, Germany). The left edge area of the defect, the middle area and the right edge area of the defect were recorded.

### 2.7. Statistical Analysis

For each experiment at least 3 biological replicates were studied. The data were tested for normality using the Shapiro–Wilk test. Values are expressed as median (min–max). Statistical analyses were performed by Mann–Whitney test or Kruskall–Wallis test followed by Dunn post-test correction. For evaluation of two factors a mixed-model analysis was done. Differences were considered statistically significant at *p* < 0.05. Analyses were performed with Prism (GraphPad Prism version 9.0).

## 3. Results

### 3.1. Tissue Preparation for Corneal Ex Vivo Culture

**Figure 1:**
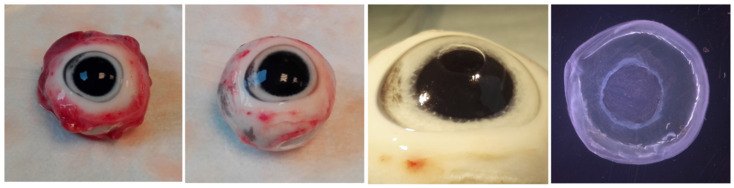
Preparation of pig eyes, wounding and corneal tissue isolation for organ culture photo documentation of the corneal preparation to start cornea organ culture. After removal of muscle and connective tissue the wound was prepared using a small biopsy punch (here 4 mm diameter) and a hockey knife. The resulting epithelial defect was visible with optimized illumination. The whole corneal tissue is excised from the eye with a 10 mm biopsy punch and was cultivated free floating in a microplate well.

Pig eyes were easily obtained at scheduled time points and in great numbers from the abattoir, so that several experiments could be performed in parallel. The experimental procedure for wound setting and preparation of corneal tissue was simple and robust. For one wounded cornea it took less than 5 min from the first step of tissue preparation until the start of ex vivo culture in a multiwell plate. Every process step could be followed with a stereomicroscope (Figure 1). For the handling it was an advantage to set the wound area with the smaller punch first and then cutting out the whole area with the 10 mm punch: (I) The scratching with a hockey knife could be carried out more precisely and reproducible, because the eyeball served as robust and stable support and (II) when positioning the 10 mm punch for cutting out, the wound was exactly placed in the middle. The excised corneal tissue sheets were cultivated free floating in a multiwell plate with the wound upward. During the culture period a swell-up of the tissue specimens was noticeable and the previously translucent corneal tissue became opaque. This effect is also known for human corneal transplants, which are often stored in a medium with dextran as deswelling agent until keratoplasty. While we used 6% dextran-500 as dehydrating additive in our media, the swelling could not be completely prevented.

### 3.2. Optical Documentation of the Corneal Defect for 72 h

**Figure 2:**
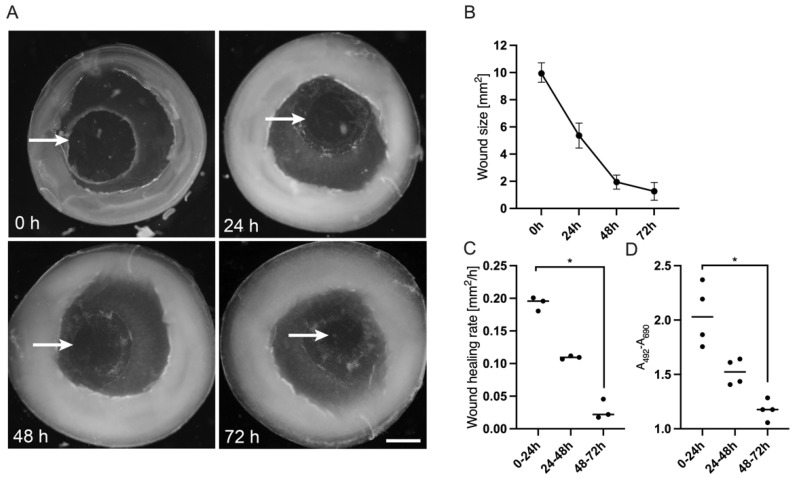
Optical documentation and analysis of wound healing in corneal organ culture model for 72 h. The wound healing process of corneas during ex vivo culture was monitored with a stereomicroscope. The specimens were cultivated in DMEM (see Table 2) for up to 72 h. (**A**) The epithelial wound margin after using a 4 mm punch could be identified at the start of the experiment (arrow, 0 h). During wound healing the margins of the newly formed epithelium could be followed visually (arrows at 24, 48 and 72 h). (**B**) The wound area was calculated at 0 h, 24 h, 48 h and 72 h and the median (range) was calculated from three specimens. (**C**) The wound healing rate was calculated for every 24 h from the start as mm^2^ per hour. D: Vitality of the corneal tissue was evaluated by measuring release of lactate dehydrogenase (LDH). The absorbance of organ culture supernatant was evaluated after 24 h, 48 h and 72 h. Scale bar measures 2 mm. Sample size *n* = 3 for (**B**,**C**), *n* = 4 for (**D**). Statistically significant differences are indicated as *: *p* < 0.05.

To establish the corneal ex vivo culture initial experiments were conducted with Dulbecco’s Modified Eagle’s Medium (DMEM), which is used for a wide range of cells or tissues. The corneal tissues were analysed by a stereomicroscope and pictures were taken every day. New epithelial tissue at the wound margin was detectable after a few hours and the area of newly formed cells enlarged with progressed cultivation period (Figure 2). The wound edges of the regenerating epithelium were clearly visible, so that the wound size was calculated at every time point. Wound size decreased significantly over 72 h from 9.94 (9.29–10.71) mm^2^ to 0.89 (0.87–2.02) mm^2^ (Figure 2B). Whether the newly formed epithelium was a result of cell divisions or cell migration had to be discussed. In this regard, a statistically significant decline of the wound healing rate over time could be seen. Initially the gap closed at a speed of 0.196 (0.180–0.201) mm^2^/h, but slowed down significantly to 0.022 (0.018–0.045) mm^2^/h during the final 24 h (Figure 2C). Correspondingly, LDH concentration in the supernatant decreased with increasing closure of the gap (Figure 2D). Following an initial peak right after inducing the trauma through trepanation LDH was reduced by almost 50% at final follow-up.

### 3.3. Histological Analysis of Corneal Wound Healing

**Figure 3:**
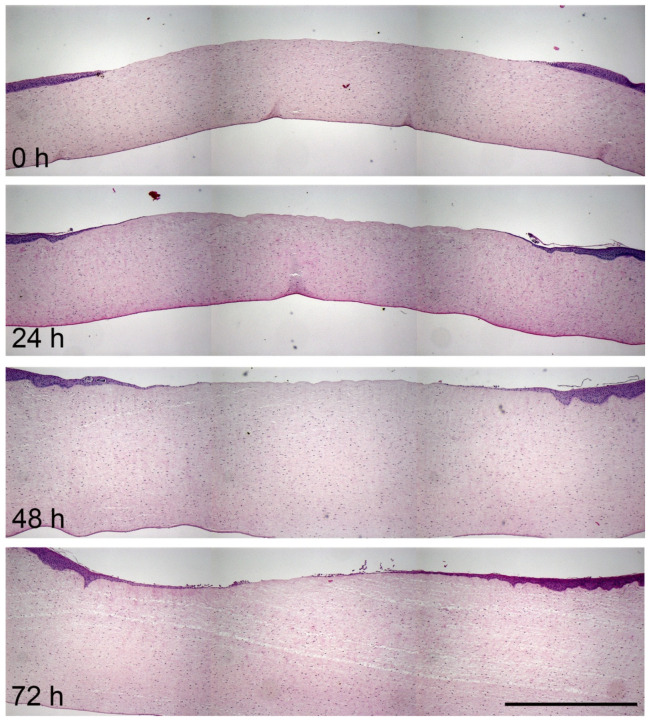
Histological documentation of the corneal wound healing process in HE-stained paraffin sections. HE-stained paraffin sections of the cornea were analysed with a stereomicroscope to follow wound healing of a 4 mm defect at day 1, 2 and 3. Thickening of the corneal stroma due to swelling processes is evident. Several single images were taken in a good magnification and via mosaic tool combined to a cornea overview. Scale bar represents 1 mm.

To get more insight into the structure and behaviour of corneal regeneration, paraffin sections were stained with haematoxylin and eosin and analysed with a stereomicroscope. The histological analysis confirmed the visually determined observations: The regeneration of the epithelial defect started at the first day with cells that migrated from the wound edge to close the gap (Figure 3). The newly formed epithelium was just 1–2 cell layers thin. The closure of the wound progressed over the cultivation period as the epithelial cells pushed themselves further and further over the corneal stroma. After 72 h a thin epithelium closed the wound.

### 3.4. Evaluation of Apoptotic Cells in the Wound Area in the Course of Cornea Ex Vivo Cultivation

**Figure 4:**
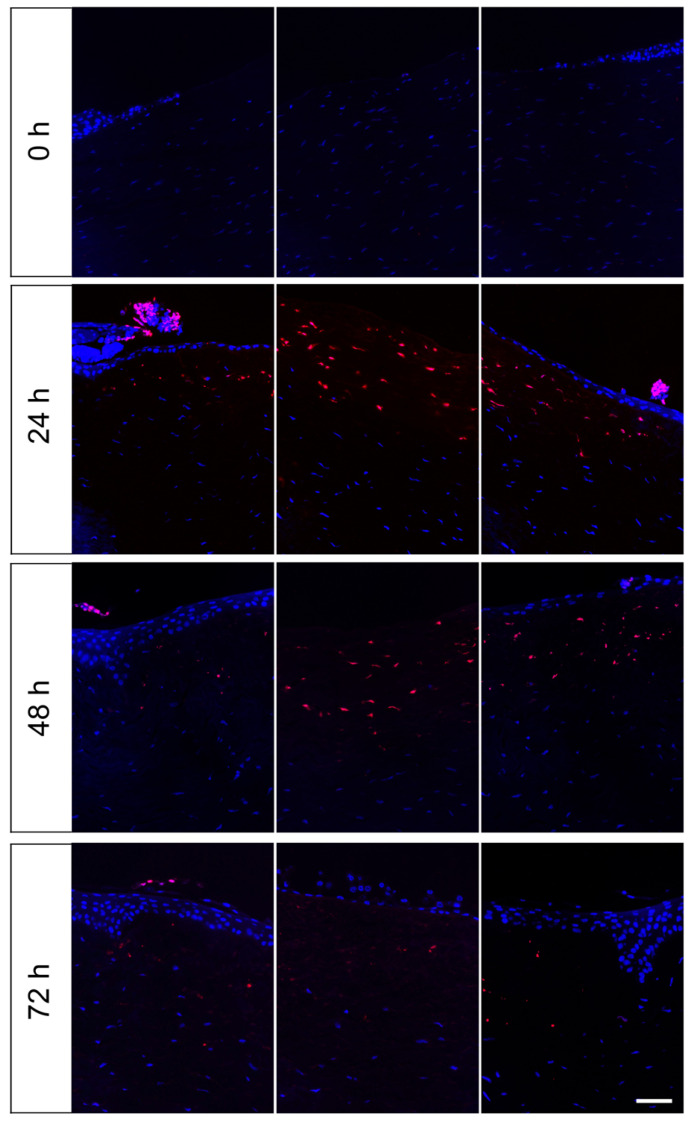
TUNEL-Assay on paraffin sections of the organ culture model after 0 h, 24 h, 48 h and 72 h. Apoptotic cells in cornea paraffin sections were determined at the start of the culture (0 h) and during ex vivo cultivation on days 1–3. Specimens had been cultured in DMEM and the lesion had a diameter of 4 mm. Images present the left wound margin (left panels), the centre of the defect (middle panels) and the right wound margin (right panels). Apoptotic cells were stained with TMR (red) and all cell nuclei were stained with DAPI (blue). Scale bar represents 50 µm.

To detect apoptosis in the wound area of the corneas, paraffin sections were prepared at the beginning of the experiment and after one, two and three days of organ culture. The sections were examined using a TUNEL staining via the “In Situ Cell Death Detection Kit”. Cells with single- and double-stranded DNA breaks that occur at the early stages of apoptosis were labelled with TMR, a red fluorescent dye. At the start of the experiment (d 0), no red-coloured and thus apoptotic cells were visible (Figure 4). On day 1, apoptotic cells were detectable at the epithelial wound margin and in the upper area of the stroma. In the stroma area beneath the defect apoptotic keratocytes were particularly numerous. On day 2, apoptotic cells were mainly detectable in the stroma. After three days only a few apoptotic cells were visible in the stroma, but none in the epithelium.

### 3.5. Influence of Defect Size and Presence of Limbus on Corneal Wound Healing

**Figure 5:**
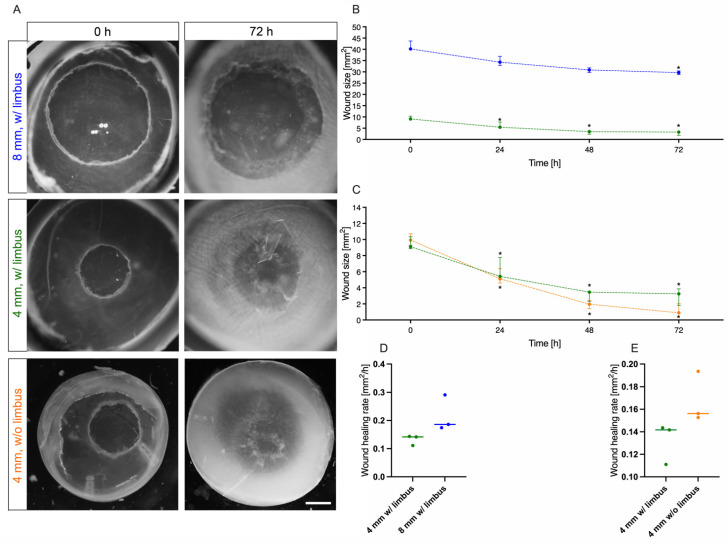
Influence of defect size and presence of limbus. For optimizing the organ culture model parameters like wound size and the presence of limbal stem cells in the limbus were evaluated. (**A**) Images were taken at 0 h and 72 h to monitor wound closure during different experimental settings. First panel (blue): Wound size 8 mm with limbus; second panel (green): Wound size 4 mm with limbus; third panel (orange): Wound size 4 mm without limbus. (**B**) Comparison of the wound closure progression of corneas with limbus for wound sizes of 4 mm (green line) and 8 mm (blue line). (**C**) Comparison of the wound closure progression of 4 mm diameter wounds of corneas with limbus (green line) and without limbus (orange line). (**D**) Wound healing rates of corneas with limbus calculated between 0 h and 48 h for wound sizes of 4 mm (green) and 8 mm (blue). (**E**) Wound healing rates of 4 mm diameter wounds calculated between 0 h and 48 h of corneas with limbus (green) and without limbus (orange). Scale bar measures 2 mm. Sample size *n* = 3. Statistically significant differences are indicated as *: *p* < 0.05.

Organ culture with living tissues is being influenced by several factors. With increasing culture time, negative effects from dying cells increase. Nevertheless, a sufficient period of time should be available to observe all cellular processes during wound healing. Here we will investigate whether the dynamics of wound healing change with epithelial erosions of different sizes. In addition to the defect of 4 mm in diameter, a defect of 8 mm was caused. Moreover, it was analysed whether the presence or absence of the limbus has an impact on corneal wound healing. Since the limbus is a reservoir for limbal stem cells, which can divide quickly in case of injury and replace the defective epithelium, the influence of this stem cell niche should be investigated. All different corneal specimens were cultured after wounding for three days and the wound size was calculated at the start of the experiment and then after 24 h, 48 h and 72 h (Figure 5A). Furthermore, the speed of wound healing was calculated by determination of the wound healing rate (mm^2^/h). First, the influence of different defect sizes with 4 and 8 mm was analysed in ex vivo cultivated corneas with limbus. Both the 4 mm and the 8 mm defects showed a significant decrease in wound sizes over 72 h. The former started at a median of 9.11 (8.86–10.35) mm^2^ and ended at 3.25 (1.81–3.87) mm^2^ (*p* = 0.014), while the latter had a median of 40.17 (39.79–43.71) mm^2^ initially and 29.68 (28.87–30.42) mm^2^ (*p* = 0.048) at final follow-up (Figure 5B). In both setups, the most dynamic passage of wound healing was seen within the first 48 h. However, during this phase healing speed was not significantly different (0.14 vs. 0.19, *p* = 0.10) between the two wound sizes (Figure 5D). As reproducibility of initial lesion area was slightly higher with the 4 mm trephine, we decided to continue the following experiments with this size. Following that, the presence of limbal stem cells and their influence on corneal wound healing was checked. The wound closure of a 4 mm defect in a cornea with limbus was compared to a cornea without limbus (Figure 5C). Even without the limbal support the wound closed significantly from 9.94 (9.29–10.71) mm^2^ at baseline to 0.89 (0.87–2.02) mm^2^ after 72 h (*p* = 0.011). After three days over 90% of the lesion was healed, but there were no statistically significant differences compared to corneas with limbus (*p* = 0.308). Since the curves nearly ran parallel the presence of the limbus seemingly did not have any significant effect at a lesion size of 4 mm. There were also no significant differences in the wound healing rate (0.14 vs. 0.16, *p* = 0.10) during the first 48 h. With the aim of establishing a robust and easy to use ex vivo model, only corneas without limbus were cultivated in the following.

### 3.6. Influence of Culture Medium on Corneal Wound Healing

**Figure 6:**
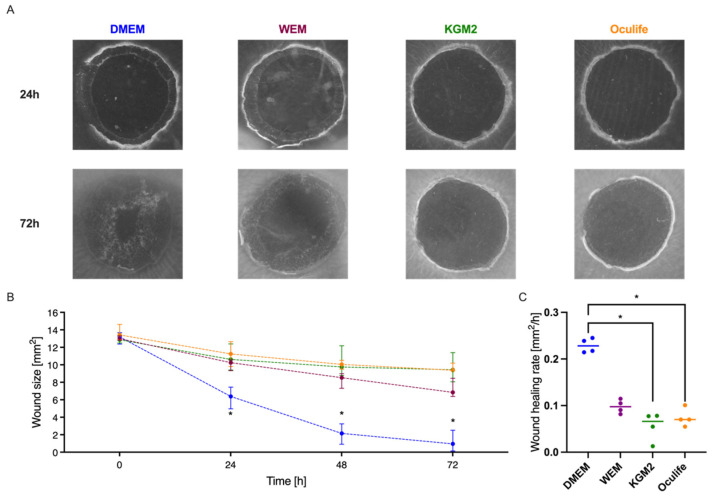
Comparison of wound healing rates in different cell culture media. The wound healing of ex vivo cultured corneas was determined evaluating the effect of different culture media. The corneal lesion was generated with a 4 mm biopsy punch. After 24 h, 48 h and 72 h of culture the wound size was calculated. (**A**) Images, which illustrate the wound healing performance were shown for 24 h and 72 h. (**B**) The tested media were Dulbecco’s modified eagle medium (DMEM, blue line), Williams E Medium (WEM, purple line), Keratinocyte Growth Medium 2 (KGM2, green line) and OcuLife (yellow line). (**C**) The wound healing rates were also calculated for the period from 0 h to 48 h. Sample size *n* = 4. Statistically significant differences are indicated as *: *p* < 0.05.

During the organ culture optimization phase, a classic serum-containing medium based on DMEM was initially used. For the in vitro culture of epithelia such as skin, there are also other special media that are considered to be beneficial for keratinocytes. Therefore, the wound healing in the cornea model was analysed in various media in order to find the optimal medium composition. During culture the wound size was determined at the start of the experiment and then 24 h, 48 h and 72 h after the lesion was generated. As shown in Figure 6, the following media were compared: William’s E Medium (WEM; purple line), a medium which is often used for skin organ culture, Keratinocyte Growth Medium 2 (KGM2; green line), which is designed for in vitro culture of epidermal keratinocytes and OcuLife (yellow line), which is optimized for the culture of human corneal epithelial cells. The application of all media resulted in a statistically significant wound size reduction. The results indicated that DMEM was superior compared to each of the other media at any time point. At final follow-up after 72 h the median lesion was 0.96 (0.14–2.52) mm^2^ for DMEM, 9.44 (8.06–9.44) mm^2^ for KGM2, 9.38 (9.35–10.19) mm^2^ for OcuLife and 6.85 (6.38–6.85) mm^2^ for WEM. However, during the dynamic phase (0–48 h) the wound healing rate of DMEM was significantly higher compared to OcuLife (*p* = 0.036) and KGM2 (*p* = 0.014), but not to WEM (*p* = 0.78).

### 3.7. Modulation of the Cornea Model with the Wound Healing Inhibitor Mitomycin

**Figure 7:**
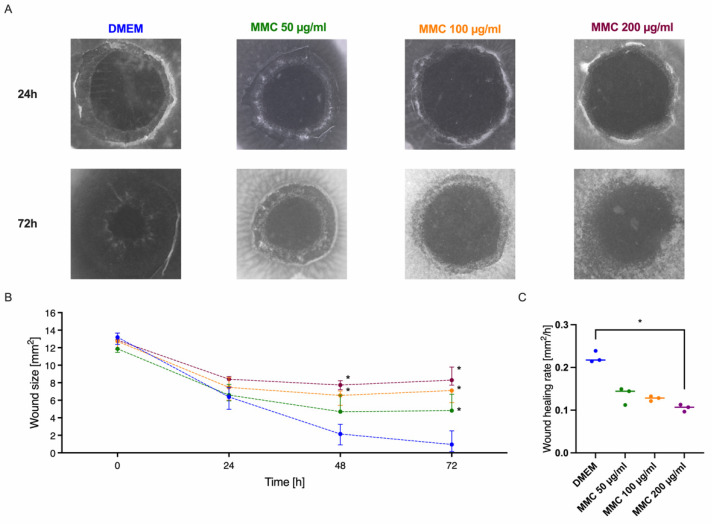
Effect of mitomycin C on corneal wound healing. The regeneration capacity of ex vivo cultured corneas was determined under the influence of the cytostatic agent mitomycin C. (**A**) Images, which illustrate the wound healing performance were shown for 24 h and 72 h. (**B**) The corneal lesion was generated with a 4 mm biopsy punch. After 24 h, 48 h and 72 h of culture the wound size was calculated. Different concentrations of mitomycin were tested: 0 µg/mL (DMEM, blue line), 50 µg/mL (green line), 100 µg/mL (orange line) and 200 µg/mL (purple line). (**C**) The wound healing rates were also calculated for the different mitomycin c concentrations evaluating the period from 0 to 48 h. Sample size *n* = 3. Statistically significant differences are indicated as *: *p* < 0.05.

For the aim to use a tissue or organ model for test purposes, it must be sensitive, so that differences in wound healing performance can be measured. Since biologic specimens are always heterogenous, standard deviations should be small to see the effects of the analysed substances. Here we analysed the influence of increasing concentrations of mitomycin C (MMC), an antimetabolite which induces cell cycle arrest, on corneal wound healing, a process were cell division and cell migration are essential. Figure 7 shows the harmful effect of MMC, which negatively affected wound healing. Nevertheless, in all specimens the wound size showed a significant decrease over 72 h regardless of the MMC concentration. Yet, comparing the MMC setups to the DMEM control a significant, slightly delayed, dose-dependent impact was evident. At final follow-up, median wound size was 4.82 (4.74–6.69) mm^2^ for MMC 50 µg/mL, 7.11 (5.74–7.33) mm^2^ for MMC 100 µg/mL and 8.30 (7.72–9.79) mm^2^ for 200 µg/mL, which were significantly larger compared to DMEM. While the wound healing rate of the samples incubated with 200 µg/mL MMC (0.11 mm^2^/h, *p* = 0.019) was significantly decreased compared to the culture in DMEM (0.22 mm^2^/h), the lower concentrations showed no significant effect on the wound healing rate.

## 4. Discussion

The use of organ culture models has several advantages and will often provide results that correspond better to the situation in vivo than in vitro models with primary cells or immortalized cell lines. Since human tissue is not regularly available and animal in vivo experiments have several ethical issues next to experimental challenges, the established organ culture model with porcine corneas appears to be the optimal alternative. First of all, the corneal architecture is maintained in those 3D models and the cells crosstalk, i.e., between keratocytes and epithelial cells remains possible. Furthermore, there is no need for elaborate equipment and since pig eyes are regularly available from the abattoir even larger experimental approaches are cost effective. In addition, this ex vivo tissue models allow the simple and easy testing of admitted exogenous factors like drugs, cytokines or even transplanted cells. Moreover, evaluation and analysis of the results is possible with quite a few procedures: Optical real time imaging of wound closure, histology of embedded cornea tissue, protein analysis of culture media, etc.

In the last few years, many research groups have already established ex vivo cornea models with animal tissues and optimized them for certain applications. Since they follow different key objectives, there are many different experimental approaches and care should be taken when comparing the results obtained.

With our model we had the aim to establish a robust and easy-to-handle ex vivo organ culture model, which allows us to study early wound healing effects, so that influences of media compositions, drugs or cell-based transplantation strategies can be analysed. Many researchers put a lot of effort into adapting the cornea culture to the in vivo situation in the best possible way. Some debate exists about the type of tissue culture: Submerged or at an air–liquid interface [7,8,9,10,11,12,13]. Richard et al. compared the ex vivo air-interface organ culture with submerged, epithelial side down cultured human corneas [7]. Together with rocking the culture plates to irrigate cornea tissues they can show a better epithelial integrity for the corneas cultured at the air–liquid interface. This approach seems to decrease intracellular edema, preserve stromal keratocytes and improve epithelial cell morphology. The system permits a long-term culture of human corneas for up to three weeks. A more recent work from Janin-Magnificat et al. followed a similar approach with air/liquid interface as well as immersed organ cultures with the difference of cultivating the submerged human corneas with the epithelial side up [14]. For laser-caused wounds they found almost no differences when comparing the expression of epithelial and wound healing specific proteins. Only the epithelial thickness was reduced to one cell layer in the submerged culture conditions compared to the air–liquid equivalents.

In the last years several groups adapt these culture systems to enable a more standardized handling and the maintenance of corneal curvature by using support structures for the cultured tissue specimens. Some groups sought to stabilize the tissue explants with the use of plastic domes from falcon tubes [9,11] or Luer Lock syringe covers [3]. Other groups use agar and collagen to fill up the corneal half shells with a gelatinous material [8,12,13,15]. Zhao and co-workers established even a sophisticated chamber system with perfusion and drop wise irrigation of rabbit corneas [10].

Our cornea 3D culture of epithelial side up, free floating cornea disks followed the approach from Janin-Magnificat et al. and is a submerged organ culture with a minimum of processing and the advantages of optimal nutritional supply and better perfusion with soluble factors [14].

The results from the lactate dehydrogenase assay, which measured cell viability, corroborate the presumption that our culture conditions were cell-protecting and preserved the viability of the corneal tissue. The initially high LDH values at the start of the culture are due to the inevitable trauma of punching out the wound and the corneal disk, but over the culture period of 72 h the LDH values and therefore cytotoxicity declined. Moreover, the results from the TUNEL assay indicate that the cultivation procedure was tissue-conserving. The apoptotic cells were mainly located in the stroma beneath the defect. The early disappearance of keratocytes after epithelial injury had been reported from others and is called “phenomenon of disappearing keratocytes”. The effect is caused by crosstalk of epithelial cells and stromal cells [16].

In addition, there was no need of adhesives to fix the tissues, i.e., with acrylate glue, which has been used by other groups to immobilize corneas and may be toxic [3,11]. Free floating corneal disks have furthermore the advantage that opacity of the cornea could be avoided, as it was observed when the tissue rests on blotting paper, agar or acrylic surfaces [9,14,17,18].

Problems like opacity and swelling of cornea tissue were also addressed in our organ culture model through addition of dextran. Dextran, a polysaccharide, increases the osmolarity of the culture medium. Such compounds are essential parts of classic ocular storage media, when tissues should be preserved for transplantation (i.e., Optisol-GS). In our ex vivo model, the addition of dextran was mandatory, because without it the corneal defect could not be located anymore due to swelling and opacification (data not shown). In the literature there is some debate about cytotoxicity of dextran, since it accumulates within the cornea [19,20,21]. However, the ex vivo culture of the cornea in dextran-containing medium for a maximum of four days is considered to be unproblematic [19].

Aiming to analyse early wound healing effects in a model, which therefore did not need to be long-term viable, we examined the effects of the presence or absence of the limbus. As the limbus is a reservoir for stem cells, this tissue between cornea and sclera has a central role in cornea regeneration. Epithelial lesions normally resolve within a couple of days in a concentric fashion due to the proliferation and migration of stem cells from the limbus or from other parts of the epithelium [14]. Comparing the corneas cultured with and without limbus there were no statistically significant differences in the general wound healing performance or the wound healing rate. These findings are in line with the work of Chang and co-workers, as they demonstrated that early re-epithelialisation after wounding is due to proliferation and migration of cells from the basal and suprabasal layers of the epithelium [22]. Corneal epithelial recovery seems to be independent of limbal epithelial stem cells at least in the first 12 h after injury as migration dominates proliferation. This suggestion is also underlined by the observation that MMC in each concentration did not change the wound healing rate within the first 24 h. Either most of the cells passed the vulnerable S and G2/M phase of the cell cycle and were at G1 stage, which is quite unlikely as cells usually are quite heterogenous in their mitosis rhythm, or migration is the major force during this initial wound healing period. We decided to use limbal-free corneal disks for further experiments, since they were easier to handle. It must be pointed out, however, that in our ex vivo model the reconstruction of a multi-layered corneal epithelium will not be possible, as no cell replenishment from the limbus is available. In fact, this is also confirmed by the histological analyses in which the regenerated epithelium remains in a single layer even three days after injury.

Regarding the defect area, no difference in the rate of wound healing could be determined between the lesion of 4 mm and 8 mm in diameter. The observation confirms studies on cultured rabbit corneas, in which the defect size also had no influence on the average rate of cellular migration during the linear healing phase. It is postulated that the epithelial cells move at a constant rate until coverage was complete and that cellular migration is the limiting step during wound healing [23]. Since the wound was closed within three days in the case of the 4 mm injuries, which was rated as a well observable healing dynamic, this defect size was used in all further investigations.

When comparing different serum-free cell culture media for the pig organ culture, the classic DMEM promoted the healing of the corneas best. Since DMEM is used for many different cell types and organisms, it seems reasonable to assume that it is generally useful in an organ culture model with different cell types, like keratinocytes, keratocytes and endothelial cells. Indeed, serum-free Minimum Essential Medium had been already used for the organ culture of human corneas, which retain vitality and remain suitable for transplantation [24]. KGM2 and OcuLife are normally used for the in vitro culture of human corneal epithelial cells or cell lines [25,26]. Their poorer performance in the wound healing model could be due to the fact that they are supportive for only one cell type. Interestingly, the WEM which was successfully established for wound healing studies in frog and human skin organ cultures [27,28] supported corneal wound healing of the pig cornea punches slightly better than KGM2 and OcuLife. In addition, it also cannot be ruled out that there are species-specific effects, so that media for human cells are not optimal suitable for pig cells. If there a specific wound healing promoting factors in DMEM or inhibitors of healing in the other media had to be analyzed in further studies.

The wound healing rates determined for the cultured pig corneas are slightly lower, but still comparable to ex vivo cultured corneas from other experimenters. Foreman et al. determined a wound healing rate for ex vivo cultured bovine corneas of 0.75 mm^2^/h for excisional wounds with a diameter of 5 mm [8]. Zhao et al. used also bovine corneas, which were cultivated in a chamber system with perfusion and irrigation [10]. They discovered different wound healing rates for their 7 mm defects according to their culture media: 0.78 mm^2^/h in serum containing media and 1.29 mm^2^/h in serum free media.

The transferability of the data gained from an animal model to humans is often a critical issue. For the selection of a suitable model, which is as close as possible to the human eye, several parameters should be considered. Among the classically used experimental animals such as rats, mice, rabbits, pigs or cattle, the eye size of pigs is closest to the human eye. In addition, the corneal thickness of pig eyes (0.68 mm) is better comparable to human eyes (0.5 mm), as other species like bovine (0.8 mm) or rabbit (0.37 mm) [15]. There are also differences in the corneal anatomical structure between the species [29]. While human and porcine corneas contain a Bowman’s layer, this layer is missing in rabbit corneas [15].

A limitation of our model is that the use of a manual trephine results in uneven wounds and cannot be reproduced identically from cornea to cornea. This can be avoided by using an excimer laser [30]. However, naturally occurring wounds are not all equivalent in depth and perfectly round. Therefore, using a trephine does actually mimic the situation in real-life more realistically. Moreover, swelling of the corneal stroma may limit the cultural duration of our model. Yet, evaluations up to five days were easily achievable, which is within the critical time frame for corneal epithelial regeneration processes. Yet, this might also vary from specimen to specimen due to baseline properties (e.g., size, thickness, endothelial cell count, stromal composition, post-mortem changes). Therefore, heterogenicity of samples could be a downside [31]. Nevertheless, the primary outcome parameter of our study (wound size) demonstrated a quite narrow range between replicates, which indicates a certain reproducibility.

In conclusion, this easy-to-handle organ culture model of pig corneas is suitable for analysing early wound healing effects. It facilitates observation of the time course and dynamic of wound closure by the use of non-invasive optical documentation with a simple stereomicroscope. The effect of substances can be studied, as we have documented for MMC, which stopped cell division and therefore hampered wound closure. Further experiments need to be performed, so that other factors like wound healing inducers or the effect of transplanted stem cells can be assessed.

## Figures and Tables

**Table 1:** Composition of culture media for corneal ex vivo cultivation.

Medium	Ingredients	Distributor
DMEM	Dulbeccos Modified Eagle Medium, high Glucose, w/L-Glutamin	Thermo Fisher Scientific GmbH, Waltham, MA, USA
	Penicillin/Streptomycin solution 1×	Merck KGaA, Darmstadt, Germany
	2.5 µg/mL Amphotericin B	Merck KGaA, Darmstadt, Germany
	6% Dextran-500	Carl Roth GmbH + Co. KG, Karlsruhe, Germany
KGM2	Keratinocyte Growth Medium 2	PromoCell, Heidelberg, Germany
	Supplement Mix including Bovine Pituitary Extract, Epidermal Growth Factor, Insulin, Hydrocortisone, Epinephrine, Transferrin, CaCl_2_	PromoCell, Heidelberg, Germany
	Penicillin/Streptomycin solution 1×	Merck KGaA, Darmstadt, Germany
	2.5 µg/mL Amphotericin B	Merck KGaA, Darmstadt, Germany
	6% Dextran-500	Carl Roth GmbH + Co. KG, Karlsruhe, Germany
OcuLife	OcuLife ™ Epithelial Basal Medium	CellSystems GmbH, Troisdorf, Germany
	OcuLife Supplements (“Life Factors”) including Insulin, Glutamin, Epinephrin, Apo-Transferrin, Extract P^TM^, Hydrocortisone, OcuFactor^TM^	CellSystems GmbH, Troisdorf, Germany
	Penicillin/Streptomycin solution 1×	Merck KGaA, Darmstadt, Germany
	2.5 µg/mL Amphotericin B	Merck KGaA, Darmstadt, Germany
	6% Dextran-500	Carl Roth GmbH + Co. KG, Karlsruhe, Germany
WEM	William’s Medium E	Merck KGaA, Darmstadt, Germany
	10 µg/mL Insulin	Sigma-Aldrich Chemie GmbH, Taufkirchen, Germany
	0.1 µg/mL Hydrocortisone	Sigma-Aldrich Chemie GmbH, Taufkirchen, Germany
	2 mM L-Glutamine	PAA Laboratories GmbH, Cölbe, Germany
	Penicillin/Streptomycin solution 1×	Merck KGaA, Darmstadt, Germany
	2.5 µg/mL Amphotericin B	Merck KGaA, Darmstadt, Germany
	6% Dextran-500	Carl Roth GmbH + Co. KG, Karlsruhe, Germany

**Table 2:** Summary of parameters for optimization and evaluation of corneal organ culture.

Influence on Test Model	Parameters
Defect size	4 mm vs. 8 mm
Role of limbus	With vs. without limbus
Organ culture medium	DMEM vs. KGM2 vs. Oculife vs. WEM
Mitomycin C as cytostatic, antiproliferative agent	50 µg/mL vs. 100 µg/mL vs. 200 µg/mL

## Data Availability

The data that support the findings of this study are available from the authors, S.S. and M.R., upon reasonable request.
